# Knowledge, awareness and practices of healthcare workers regarding antimicrobial use, resistance and stewardship in Zambia: a multi-facility cross-sectional study

**DOI:** 10.1093/jacamr/dlae076

**Published:** 2024-05-17

**Authors:** Steward Mudenda, Billy Chabalenge, Victor Daka, Elimas Jere, Israel Abebrese Sefah, Evelyn Wesangula, Kaunda Yamba, Julian Nyamupachitu, Nathan Mugenyi, Zia Ul Mustafa, Mirfin Mpundu, Joseph Chizimu, Roma Chilengi

**Affiliations:** Department of Pharmacy, School of Health Sciences, University of Zambia, Lusaka, Zambia; Department of Medicines Control, Zambia Medicines Regulatory Authority, Lusaka, Zambia; Department of Public Health, Michael Chilufya Sata School of Medicine, Copperbelt University, Ndola, Zambia; Department of Medicines Control, Zambia Medicines Regulatory Authority, Lusaka, Zambia; Pharmacy Practice Department, School of Pharmacy, University of Health and Allied Sciences, Volta Region, PMB 31, Ho, Ghana; Strengthening Pandemic Preparedness, Eastern, Central, and Southern Africa Health Community, Arusha, Tanzania; Antimicrobial Resistance Coordinating Committee, Zambia National Public Health Institute, Lusaka, Zambia; Action on Antibiotic Resistance (ReAct) Africa, Lusaka, Zambia; Faculty of Medicine, Mbarara University of Science and Technology, Mbarara, Uganda; Discipline of Clinical Pharmacy, School of Pharmaceutical Sciences, Universiti Sains Malaysia, Gelugor, Penang, 11800, Malaysia; Department of Pharmacy Services, District Headquarter (DHQ) Hospital, Pakpattan, 57400, Pakistan; Action on Antibiotic Resistance (ReAct) Africa, Lusaka, Zambia; Antimicrobial Resistance Coordinating Committee, Zambia National Public Health Institute, Lusaka, Zambia; Antimicrobial Resistance Coordinating Committee, Zambia National Public Health Institute, Lusaka, Zambia

## Abstract

**Background:**

Antimicrobial resistance (AMR) poses a threat to public health globally. Despite its consequences, there is little information about the knowledge, awareness, and practices towards AMR among healthcare workers (HCWs). Therefore, this study assessed the knowledge, awareness and practices regarding antimicrobial use (AMU), AMR and antimicrobial stewardship (AMS) among HCWs who are involved in the implementation of AMS activities across eight hospitals in Zambia.

**Methods:**

A cross-sectional study was conducted among 64 HCWs from October to December 2023 using a semi-structured questionnaire. Data were analysed using IBM SPSS version 25.0.

**Results:**

Of the 64 HCWs, 59.4% were females, 60.9% were aged between 25 and 34 years, 37.5% were nurses, 18.7% were pharmacists, 17.2% were medical doctors and only one was a microbiologist. Overall, 75% of the HCWs had good knowledge, 84% were highly aware and 84% had good practices regarding AMU, AMR and AMS. Most of the HCWs (90.6%) responded that they had a multidisciplinary AMS team at their hospitals and were implementing the use of the WHO AWaRe classification of antibiotics.

**Conclusion:**

This study found good knowledge levels, high awareness and good practices regarding AMU, AMR and AMS among HCWs who were involved in the implementation of AMS activities in hospitals in Zambia. Additionally, most hospitals have been conducting AMS training and implementing the use of the WHO AWaRe classification of antibiotics. However, there is still a need to address some identified gaps in AMU and AMR through the strengthening of AMS activities in hospitals.

## Introduction

Antimicrobial resistance (AMR) is a global public health problem that has been worsened by the inappropriate use of antimicrobials in humans, animals, agriculture and the environment.^1–4^ Consequently, AMR has become a growing problem globally and a threat to global public health with many consequences including prolonged hospital stay, increase in mortality and economic burdens.^1,2,5–8^ Antimicrobials are widely used in the human health sector, and this has contributed to the emergence of AMR.^1,9–11^ Healthcare workers (HCWs) are highly responsible for the use of antimicrobials in the human healthcare sector particularly in a hospitalized setting.^12^ Hence, it is critical to understand the knowledge, awareness, and practices of HCWs regarding antimicrobial use (AMU), AMR, and antimicrobial stewardship (AMS) in hospitals, as reported in other studies.^13–15^

The drivers of AMR in the human healthcare sector are quite complex.^16–20^ Evidence has demonstrated that the overuse and misuse of antimicrobials is a major contributing factor to AMR in humans.^19,21–24^ The lack of knowledge and awareness of AMU, AMR and AMS among HCWs is also a contributing factor to the development of antimicrobial-resistant infections.^15,25^ This is because HCWs may inappropriately prescribe, dispense and administer antimicrobials. In Low- and middle-income countries (LMICs), drivers of AMR are complex and include a lack of diagnostic tools, access to antimicrobials without prescriptions, shortage of medicines, shortage of HCWs, inappropriate use of antibiotics in animals, poor hygiene and sanitation, weak regulatory systems to restrict access to antimicrobials, poor clinical care and a lack of robust AMR surveillance programmes.^26–30^ Additionally, the presence of poor-quality antimicrobials also contributes to AMR in the LMICs.^31,32^ Alongside this, the inappropriate use of antimicrobials in clinical settings is among the major contributors to AMR.^7,20,33^

AMS programmes are essential in combating AMR and its associated factors.^12,34–36^ All AMS programmes must meet the core elements including leadership commitment, accountability and responsibilities, availability of expertise on infection prevention and control (IPC), AMS actions, education and training, monitoring and surveillance of AMU and AMR, and reporting feedback regarding AMS results to the AMS team.^12,37–40^ Instigating AMS programmes in hospitals ensures that antimicrobials are used rationally, for the right patient, correct dose, time and duration of therapy through appropriate route of administration.^41^ Additionally, AMS may help improve HCW’s awareness, knowledge, attitudes and practices towards AMR and the use of antimicrobials.^42,43^ Previous studies have shown that the introduction of AMS programmes in hospitals improves the awareness and knowledge of AMU, AMR and AMS leading to improved prescribing practices and use of antimicrobials.^44–46^ Hence, implementation of effective AMS programmes in hospitals is very critical in promoting the rational use of antimicrobials.^46,47^

In May 2015, the WHO developed a Global Action Plan (GAP) on AMR and emphasized the importance of education in addressing AMR. The Quadripartite (WHO, World Organization for Animal Health, United Nations Environment Programme and the Food and Agriculture Organization of the UN) have been spearheading the implementation of the GAP on AMR.^48^ In addition, WHO member countries were encouraged to develop National Action Plans (NAPs) on AMR to address this global problem.^48,49^ Furthermore, countries were encouraged to heighten their surveillance of infections and AMR.^48,50^ The GAP on AMR promotes tackling AMR using a One Health approach.^48^ Further, the GAP and NAPs on AMR aim at increasing the awareness and knowledge of AMU, AMR and AMS among various communities including HCWs.^28,48,51–54^ This would in turn promote the appropriate use of antimicrobials and reduce the emergence and spread of AMR.^28^

Zambia is a country in the sub-Saharan African region that has reported on the factors that contribute to AMR.^29,55–60^ Additionally, many microorganisms have demonstrated resistance to most antimicrobials used in the healthcare system in Zambia.^61–76^ Some studies have reported inappropriate prescribing patterns of antibiotics in public sector hospitals.^60,77–82^ In response to the GAP on AMR, Zambia, a member state of the WHO, developed a NAP on AMR to address antimicrobial-resistant infections.^52,83^ Additionally, the Antimicrobial Resistance Coordinating Committee (AMRCC) of the Zambia National Public Health Institute (ZNPHI) implements and monitors the activities of the Zambian NAP on AMR.^52,84^ However, there is a paucity of information on the knowledge, attitudes and practices regarding AMR and AMS among HCWs in public hospitals. There is a paucity of information regarding the knowledge, attitudes and practices regarding AMU, AMR and AMS among public sector HCWs in Zambia, as evidenced by a few published studies before the establishment and implementation of AMS programmes.^85,86^ Therefore, this study was conducted to assess the knowledge, awareness and practices of HCWs on AMU, AMR and AMS across eight selected public hospitals in Zambia.

## Materials and methods

### Study design, population and period

**Figure 1.**
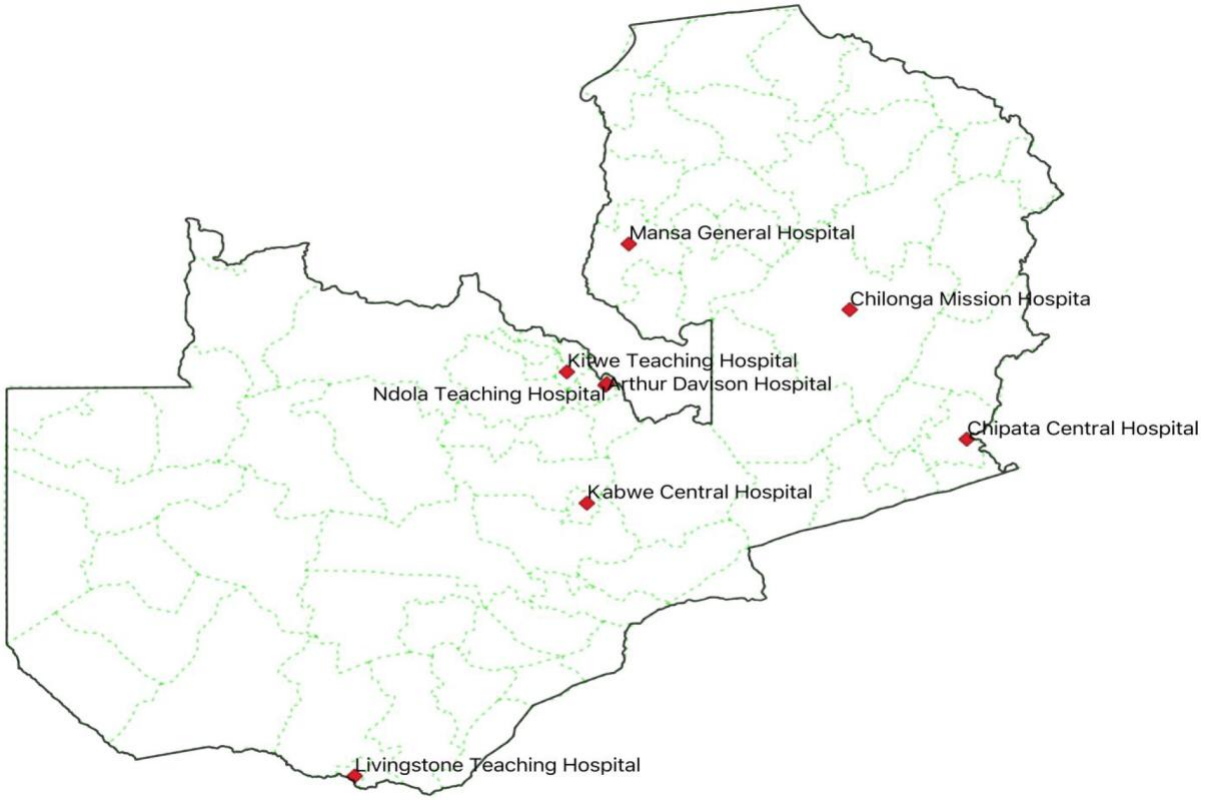
Map of Zambia indicating the surveyed hospitals.

A cross-sectional survey was conducted among HCWs across eight hospitals in selected districts of Zambia from October 2023 to December 2023 in eight provinces. The eight hospitals included five tertiary-level hospitals including Arthur Davison Children’s Hospital (Copperbelt Province), Chipata Central Hospital (Eastern Province), Kitwe Teaching Hospital (Copperbelt Province), Livingstone Teaching Hospital (Southern Province), Ndola Teaching Hospital (Copperbelt Province) and three were secondary level hospitals that included Chilonga Mission Hospital (Muchinga Province), Kabwe Central Hospital (Central Province) and Mansa General Hospital (Luapula Province) (Figure 1). In Zambia, all the hospitals are required to have a multidisciplinary AMS committee composed of medical doctors, infectious disease specialist physicians, pharmacists, nurses, microbiologists, biomedical scientists, environmental health experts and public health experts to manage AMS and implement IPC activities. The AMS committee reports directly to the Medicines and Therapeutics Committee of the hospital. The eight hospitals were chosen because they were the first cohort in which AMS programmes were established by the AMRCC of the ZNPHI, with future expansion of AMS programmes in all the hospitals in Zambia. Additionally, the selected hospitals happen to be referral hospitals in these particular provinces. Therefore, the study enrolled the HCWs who were trained in AMU, AMR and AMS and were involved in the implementation of AMS activities as enshrined in the Zambian NAP on AMR.

### Sample size estimation and sampling criteria

The sample size was determined using the sample size for cross-sectional studies.^87^ The hospital multidisciplinary AMS teams are composed of an average of nine members giving a total population of 72. We estimated a minimum required sample size of 61 participants using a conservative estimate of 50%, precision of 5%, and a 95% confidence interval and extrapolating to a finite population of 72. A total of 64 participants were enrolled, with eight HCWs selected per hospital. To obtain clear information regarding AMU, AMR and AMS, only HCWs who were directly involved in the implementation of the hospital-based AMS programmes in the selected hospitals were approached and enrolled in the study. Thus, the purposive sampling method ensures that the data collected is better matched to the aims and objectives of the study, thereby improving the trustworthiness of the data and findings and the rigour of the survey.^88^ Additionally, this method promotes the dependability, transferability, credibility and confirmability of the findings.^88^

### Data collection

The data were collected using an adapted tool from a previous study.^46^ The questionnaire had five sections including (i) socio-demographics of HCWs, (ii) knowledge of HCWs on antimicrobials and their use, (iii) awareness of HCWs on AMR and AMS, (iv) hospital practice regarding the use of antimicrobials and (v) the open-ended questions on AMS teams (a lead person from each hospital was asked on the composition of the AMS team at the facility) and implementation of the AWaRe classification of antibiotics. For the knowledge, awareness and practice questions, there were five responses provided including agreed (A), disagreed (D), neutral (N), strongly agreed (SA) and strongly disagreed (D). Data collection was done by eight data collectors who visited the eight hospitals and administered a semi-structured questionnaire to the HCWs who were directly involved in the implementation of the hospital-based AMS programmes.

### Data analysis

The data collected were analysed using Statistical Packaging for Social Sciences (SPSS) version 25.0. The frequencies and percentages were reported in categorical variables. Scores/codes were allocated as 1 for SD, 2 for D, 3 for N, 4 for A and 5 for SA. Reverse coding was used for negatively worded questions, i.e. SA = 1, A = 2, N = 3, D = 4 and SD = 5. To report the results, agreed and strongly agreed were reported as agreed, whereas disagreed and strongly disagreed were reported as disagreed. Knowledge had a total of 11 questions translating into a minimum of 11 scores and a total of 55 scores. Awareness had 10 questions translating into a minimum of 10 scores and a total of 50 scores. Practice had a total of 13 questions translating into a minimum of 13 scores and a maximum of 65 scores. Participants who had good knowledge, awareness and practice towards AMU, AMR and AMS scored 80% and above, as reported in earlier studies.^85,89^ Therefore, good knowledge had scores of 44 and above, high awareness had scores of 40 and above, and good practices had scores of 56 and above. Consequently, participants who scored below 80% were classified as having poor knowledge, awareness and practice AMU, AMR and AMS.

### Ethical approval

Before conducting the study, we obtained ethical approval from the Tropical Diseases Research Centre Ethics Committee with an approval number of TRC/C4/09/2023. All the participants were informed about the purpose of the study. Participation was voluntary after providing informed and written consent.

## Results

This study enrolled 64 HCWs including 38 (59.4%) females and most (60.9%) were aged 25 to 34 years. Most of the participants (37.5%) were nurses and 32.8% had worked for 1 to 5 years as presented in Table 1.

**Table 1.** Socio-demographic characteristics of healthcare workers

Variable	Characteristic	Frequency (%)
Gender	Female	38 (59.4)
	Male	26 (40.6)
Age (years)	19–24	2 (3.1)
	25–34	39 (60.9)
	35–44	19 (29.7)
	45–54	4 (6.3)
Profession	Biomedical scientists	9 (14.1)
	Clinical officers	2 (3.1)
	Environmental health personnel	3 (4.7)
	Medical doctors	11 (17.2)
	Medical licentiate	1 (1.6)
	Microbiologist	1 (1.6)
	Nurses	24 (37.5)
	Pharmacy personnel	12 (18.7)
	Physiotherapy technologist	1 (1.6)
Years of work experience	<1	5 (7.8)
	1–5	21 (32.8)
	6–10	18 (28.1)
	>10	20 (31.3)
Years of working in the current facility	<1	9 (14.1)
	1–5	21 (32.8)
	6–10	18 (28.1)
	>10	16 (26.0)

Our study found that most HCWs (59.4%) disagreed that antibiotics are used in the management of all infections and 92.2% disagreed that they should be stopped once someone felt better. Additionally, most HCWs (93.7%) agreed that the frequent use of antibiotics may decrease their efficacy of treatment and 96.9% agreed that their use must be strictly controlled. Additionally, 84.4% and 90.9% of the HCWs agreed that the prescriber’s skills and knowledge and the patient’s self-medication practices contribute to the inappropriate use of antibiotics (Table 2).

**Table 2.** Knowledge of antibiotics and their use among healthcare workers

Knowledge statements	A, *n* (%)	D, *n* (%)	N, *n* (%)	SA, *n* (%)	SD, *n* (%)
Antibiotics are used in the management of all infections	11 (17.2)	17 (26.6)	4 (6.3)	11 (17.2)	21 (32.8)
Treatment with antibiotics should be stopped once you feel better, especially the expensive ones	1 (1.6)	15 (23.4)	2 (3.1)	2 (3.1)	44 (68.8)
It’s okay to use antibiotics that were given to a friend or family member, as long as they were used to treat the same illness	0 (0.0)	11 (17.2)	0 (0.0)	1 (1.6)	52 (81.3)
It’s okay to buy the same antibiotics, or request these from a doctor if you're sick and they helped you get better when you had the same symptoms before	0 (0.0)	15 (23.4)	2 (3.1)	1 (1.6)	46 (71.9)
Frequent use of antibiotics may decrease the efficacy of treatment	23 (35.9)	3 (4.7)	0 (0.0)	37 (57.8)	1 (1.6)
Antibiotic use should be strictly controlled	17 (26.6)	0 (0.0)	1 (1.6)	45 (70.3)	1 (1.6)
It is possible for the antibiotics we are using today to stop working properly in the future	16 (25.0)	3 (4.7)	1 (1.6)	44 (68.8)	0 (0.0)
Counselling of patients may influence the inappropriate use of antibiotics	13 (20.3)	11 (17.2)	2 (3.1)	27 (42.2)	11 (17.2)
Skills and knowledge of prescribers may influence the inappropriate use of antibiotics	18 (28.1)	5 (7.8)	0 (0.0)	36 (56.3)	5 (7.8)
Patient self-medication influences inappropriate use of antibiotics	17 (26.6)	2 (3.1)	1 (1.6)	41 (64.1)	3 (4.7)
Inadequate supervision of the medicine administration may influence the inappropriate use of antibiotics	25 (39.1)	2 (3.1)	1 (1.6)	35 (54.7)	1 (1.6)

**Figure 2.**
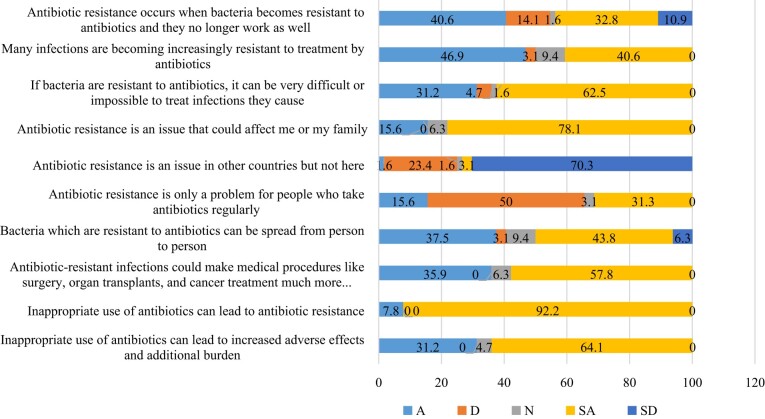
Participants’ awareness of AMR and stewardship.

This study found that most HCWs (73.4%) knew that AMR occurs when bacteria become resistant to antibiotics. Further, 87.5% of the HCWs agreed that many infections are becoming increasingly resistant to antibiotics. Furthermore, 93.8% agreed that it is difficult or impossible to treat infections caused by antibiotic-resistant bacteria (Figure 2). Intriguingly, 93.7% of the HCWs disagreed that AMR is a problem in other countries and not here in Zambia. Additionally, 95.3% of the HCWs agreed that inappropriate use of antibiotics can lead to increased adverse effects and additional burdens (Figure 2).

Our study found that most of the HCWs agreed that a patient’s clinical condition (79.7%) and microbiological results in symptomatic patients (85.9%) influence the decision to start a patient on antimicrobial therapy in the hospital. Further, most HCWs agreed that drivers of AMR include the inappropriate prescribing habits of antibiotics (89%), a lack of effective diagnostics tools to diagnose bacterial infections (89%), patients’ self-medication with antibiotics without consulting healthcare professionals (98.4%) and spread of bacteria in healthcare settings due to poor hygiene practices (84.4%). Finally, most HCWs agreed that controlling AMR should involve consulting infectious diseases experts (98.4%), obtaining local antibiotic resistance profiles (85.3%), targeting antimicrobial therapy to likely pathogens (100%) and changing the attitudes of prescribers and patients to reduce unnecessary antibiotic usage (98.4%) (Table 3).

**Table 3.** Hospital practices of participants regarding the use of antimicrobials

Statement	Characteristic	A	D	N	SA	SD
The following factors influence the decision to start a patient on antimicrobial therapy in the hospital	The patient's clinical condition	27 (42.2)	7 (10.9)	4 (6.3)	24 (37.5)	2 (3.1)
	Microbiological results in symptomatic patients	16 (25.0)	2 (3.1)	6 (9.4)	39 (60.9)	1 (1.6)
The following practices contribute to AMR in hospitals	Inappropriate prescribing habits of antibiotics	10 (15.6)	1 (1.6)	5 (7.8)	47 (73.4)	1 (1.6)
	Lack of effective diagnostics tools to diagnose bacterial infections	18 (28.1)	3 (4.7)	4 (6.3)	39 (60.9)	0 (0.0)
	Patients’ self-medication with antibiotics without consulting healthcare professionals	9 (14.0)	1 (1.6)	0 (0.0)	54 (84.4)	0 (0.0)
	The spread of bacteria in healthcare settings due to poor hygiene practices	24 (37.5)	2 (3.1)	8 (12.5)	30 (46.9)	0 (0.0)
Antibiotic prescribing in healthcare facilities	Antibiotics are overprescribed in this facility	30 (46.9)	5 (7.8)	4 (6.3)	23 (35.9)	2 (3.1)
	Antibiotic choices should only be made based on laboratory results always	20 (31.3)	12 (18.8)	13 (20.3)	16 (25.0)	3 (4.7)
	Current antibiotics available in the facility are unable to treat some infections	34 (53.1)	9 (14.1)	10 (15.6)	7 (10.9)	4 (6.3)
	There are policies and protocols for antibiotic use in this facility	24 (37.5)	16 (25.0)	17 (26.6)	4 (6.3)	3 (4.7)
The following practices may help control antimicrobial resistance	Consulting with infectious diseases experts	34 (53.1)	1 (1.6)	0 (0.0)	29 (45.3)	0 (0.0)
	Obtaining local antibiotic resistance profile	24 (37.5)	1 (1.6)	2 (3.1)	37 (57.8)	0 (0.0)
	Targeting antimicrobial therapy to likely pathogens	24 (37.5)	0 (0.0)	0 (0.0)	40 (62.5)	0 (0.0)
	Changing the attitudes of prescribers and patients to reduce unnecessary antibiotic usage	12 (18.7)	0 (0.0)	1 (1.6)	51 (79.7)	0 (0.0)

**Figure 3.**
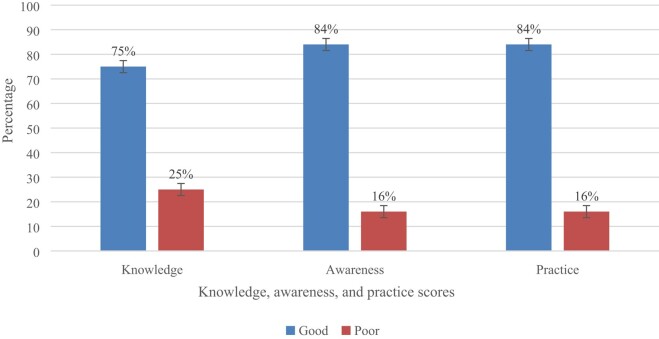
Overall scores of knowledge, awareness and practices regarding AMU, AMR and AMS among healthcare workers.

This study found that most (75%) of the HCWs scored above the cut-off point of 80% and hence had good knowledge of AMU. Further, 84% of the HCWs were aware of AMR and 84% had good practices towards AMR and AMS as they scored above the 80% cut-off point of good KAP (Figure 3).

### Antimicrobial stewardship team availability and membership composition

Notably, 58/64 (90.6%) of the HCWs mentioned that they had an AMS team in their hospital and that the teams were implementing the use of the WHO AWaRe classification of antibiotics. Regarding the AMS team composition at the facility, the lead people gave the responses as follows; Participant number 1 said that the AMS team was composed of medical doctors, nurses, pharmacists and biomedical/laboratory scientists. Participant number 2 responded that the AMS team was made up of medical doctors, nurses, pharmacists, biomedical/laboratory scientists, clinical officers and a nutritionist. Participant number 3 stated that the AMS team in their hospital was made up of medical doctors, nurses, pharmacists, biomedical/laboratory scientists and an environmental health technologist. Participant number 4 responded that their AMS team comprised medical doctors, nurses, pharmacists, biomedical/laboratory scientists, public health officers and an information technologist officer. Participant number 5 stated that the AMS team in their hospital was made up of medical doctors, nurses, pharmacy technologists, pharmacists, biomedical/laboratory scientists, clinical officer general and microbiologists. Participant number 6 stated that their AMS team comprised a medical doctor, physician assistant, nurse, pharmacy technologist, pharmacist, biomedical/laboratory scientist and physiotherapy technologist. Participant number 7 responded that the AMS team was made up of medical doctors, nurses, pharmacists, biomedical/laboratory scientists, clinical officers and a nutritionist. Participant number 8 stated that the AMS team in their hospital was made up of medical doctors, nurses, pharmacists, biomedical/laboratory scientists and an environmental health technologist. Of the total participants, 58 (87.5%) stated that minutes were always written each time they had their monthly, quarterly, bi-annual and annual AMS team meetings.

## Discussion

To the best of our knowledge, this is the first study to assess the knowledge, awareness and practices regarding AMU, AMR and AMS among HCWs in Zambia. We found good knowledge, high awareness and good practices regarding AMU, AMR and AMS among HCWs in Zambia. These findings could be due to the exposure of HCWs to education and training through the ongoing implementation of AMS programmes in selected hospitals in Zambia.

It is well established that a lack of knowledge among HCWs on AMR and AMS can result in the inappropriate use of antimicrobials.^25^ Zambia is currently implementing its 2017–2027 Multi-Sectoral NAP on AMR with the first objective focusing on raising awareness, educating and training HCWs to optimize AMU.^52^ As a result of this, many HCWs especially in secondary and tertiary hospitals have undergone training in AMS. It is highly recognized that the creation of strong AMS programmes in hospitals is important for the promotion of appropriate use of antimicrobials.^41,90–92^

Our study found that 75% of the HCWs had good knowledge of AMU. These findings are in line with those reported in other studies. Most HCWs in our study knew that antibiotics are not used in the management of all infections. Additionally, most of the HCWs knew that is possible for the antibiotics we are using today to stop working properly in the future. Furthermore, the HCWs knew that inadequate supervision of the medicine administration may contribute to the inappropriate use of antibiotics. The good knowledge of HCWs regarding AMU and AMR could be due to their involvement in the training and implementation of AMS programmes in their hospitals. Similar findings have been reported in which AMS programmes improved the knowledge of HCWs on AMU and AMR.^93^ Our findings are similar to other studies that also reported that most of the HCWs had good knowledge of AMR and AMS.^46,94,95^ Education and training through AMS activities promote the knowledge of HCWs on AMU and promotes rational prescribing of antimicrobials.^90^ Conversely, our findings are in contrast to those reported in other studies where HCWs had low or poor knowledge regarding AMU, AMR and AMS.^13,43,96^ Therefore, our findings and those reported by others revealed discrepancies in the knowledge of HCWs on AMR with some researchers reporting good knowledge while others reporting poor knowledge of AMU, AMR and AMS. This underscores the need to establish and strengthen AMS programmes in hospitals to increase the knowledge of HCWs on AMR and promote the rational use of antimicrobials.^38,93,97–101^

The present study found that most HCWs in Zambia who were involved in the implementation of AMS were aware of AMU, AMR and AMS. The current study found that most of the HCWs were aware that antibiotic resistance occurs when bacteria become resistant to antibiotics and that this problem affects everyone, including other countries. Consequently, they felt that antimicrobial-resistant bacteria can be transmitted from one person to another. Alongside this, the HCWs were aware that the inappropriate use of antibiotics is among the main drivers of AMR. Our findings corroborate reports from other studies where most HCWs were aware of AMR.^102,103^ The high awareness of AMR among our study participants could be due to the instigated AMS programmes in the surveyed hospitals, alongside their experience during practice. In contrast to our findings, some studies found that few HCWs were aware of AMR and AMS practices.^46,96,104^ This could be due to a lack of implementation of education and training on AMR and AMS in their hospitals.^105^ In Nigeria, a study reported that the low awareness about AMU and AMR was due to a lack of established and strengthened AMS programmes in some surveyed tertiary hospitals.^96^ In this regard, we believe continuous education and training on AMU, AMR and AMS may improve the awareness and use of antimicrobials among HCWs.

The current study found that most of the HCWs in Zambia had good practices towards AMS. Our study revealed that most of the HCWs knew that the inappropriate prescribing habits of antibiotics in hospitals and a lack of effective diagnostics tools to diagnose bacterial infections contribute to the emergence and spread of AMR. Consequently, the HCWs felt that the current antibiotics available in hospitals are unable to treat some infections. Intriguingly, the HCWs felt that addressing AMR would require obtaining local antibiotic resistance profiles, consulting with infectious diseases experts, providing targeted antimicrobial therapy to likely pathogens and changing the attitudes of prescribers and patients to reduce unnecessary use of antibiotics. The good practices that were reported in our study could be due to the AMS activities that have been implemented in the selected sites and show that AMS interventions are critical in promoting the rational use of antibiotics and addressing AMR. Contrary to our findings, some studies reported poor practices towards AMR and AMS among HCWs including studies done in Ghana,^13^ Nigeria,^96^ and the Kingdom of Saudi Arabia,^106^ respectively. Our study revealed a higher practice score than the one that was reported in Uganda.^107^ The poor practices could be due to a lack of AMS programmes in some hospitals.^96^ Therefore, based on evidence-based studies, we believe that the establishment of AMS in hospitals improves practices regarding the use of antibiotics and leads to an improvement in prescribing practices and a reduction in the misuse and overuse of antibiotics.^44,45,108^

Our study found that most of the hospitals surveyed had multidisciplinary AMS teams in place including critical members such as physicians, pharmacists, nurses and microbiologists. However, we noted that there were few or no microbiologists in most of the AMS teams. The presence of multidisciplinary AMS teams in hospitals is very important because AMR must be addressed using a collaborative-multidisciplinary approach.^34^ Consequently, the absence of microbiologists in most AMS teams may affect the quality of microbiology results reported from the laboratories. The absence of critical members of the AMS teams has been reported in other studies and may affect the goals of AMS programmes.^109,110^ The AMS teams are critical because they are responsible for planning and implementing strategies to achieve the set goals towards improving the appropriate use of antibiotics.^99^ Subsequently, AMS teams promote the need to use recommended protocols when prescribing antibiotics.^111,112^ Implementation of AMS programmes by the AMS teams in hospitals has been reported to reduce the consumption of antibiotics, increase adherence to treatment guidelines, provide feedback on antibiotic prescribing practices and improve patient outcomes.^108,113–118^ In this regard, we emphasize the need to promote the establishment and strengthening of multidisciplinary AMS teams in healthcare facilities, similar to recommendations from other studies.^119,120^

We are aware that our study had limitations. One of the constraints of this study was that only a small number of HCWs from the hospital under study participated. However, the fact that the study subjects are directly involved in AMS activities and exhibited good knowledge, awareness and practice shows the impact of having multidisciplinary AMS teams in all hospitals to champion the AMS programmes.

## Conclusion

This study found good knowledge, high awareness and good practices regarding AMU, AMR and AMS among HCWs in Zambia. The findings of a good KAP could be because of the implementation of AMS training in the surveyed hospitals indicating the importance of educational interventions. However, there is still a need to address some identified gaps in some areas such as knowledge of AMU and AMR through the strengthening of AMS activities in hospitals. This is because we observed slightly lower knowledge scores compared to the awareness and practice scores. Moreover, ongoing monitoring and evaluation of AMS programmes are crucial to assess their effectiveness and identify areas for further enhancement. By prioritizing continuous education, capacity-building and quality improvement initiatives, healthcare facilities in Zambia can further reinforce their commitment to combating AMR and promoting prudent AMU, ultimately safeguarding both individual patient health and public health at large.
