# Correction to: Knowledge, awareness and practices of healthcare workers regarding antimicrobial use, resistance and stewardship in Zambia: a multi-facility cross-sectional study

**DOI:** 10.1093/jacamr/dlae106

**Published:** 2024-07-03

This is a correction to: Steward Mudenda, Billy Chabalenge, Victor Daka, Elimas Jere, Israel Abebrese Sefah, Evelyn Wesangula, Kaunda Yamba, Julian Nyamupachitu, Nathan Mugenyi, Zia Ul Mustafa, Mirfin Mpundu, Joseph Chizimu, Roma Chilengi, Knowledge, awareness and practices of healthcare workers regarding antimicrobial use, resistance and stewardship in Zambia: a multi-facility cross-sectional study, *JAC-Antimicrobial Resistance*, Volume 6, Issue 3, June 2024, dlae076, https://doi.org/10.1093/jacamr/dlae076

This article has been updated to correct an error in author Israel Abebrese Sefah's name.

